# Simultaneous detection of α-Lactoalbumin, β-Lactoglobulin and Lactoferrin in milk by Visualized Microarray

**DOI:** 10.1186/s12896-017-0387-9

**Published:** 2017-09-12

**Authors:** Zhoumin Li, Fang Wen, Zhonghui Li, Nan Zheng, Jindou Jiang, Danke Xu

**Affiliations:** 1 State Key Laboratory of Analytical Chemistry for Life Science, School of Chemistry and Chemical Engineering, Nanjing University, Nanjing, 210093 China; 2 School of Chemistry and Biological Science, Nanjing University Jingling College, Nanjing, 210089 China; 3 Ministry of Agriculture-Key Laboratory of Quality & Safety Control for Milk and Dairy Products, Institute of Animal Science, Chinese Academy of Agricultural Sciences, Beijing, 100193 People’s Republic of China; 4 Ministry of Agriculture Dairy Quality Supervision and Testing Center, Harbin, 150090 China

**Keywords:** Visualized microarray, α-Lactoalbumin, β-Lactoglobulin, Lactoferrin

## Abstract

**Background:**

α-Lactalbumin (a-LA), β-lactoglobulin (β-LG) and lactoferrin (LF) are of high nutritional value which have made ingredients of choice in the formulation of modern foods and beverages. There remains an urgent need to develop novel biosensing methods for quantification featuring reduced cost, improved sensitivity, selectivity and more rapid response, especially for simultaneous detection of multiple whey proteins.

**Results:**

A novel visualized microarray method was developed for the determination of a-LA, β-LG and LF in milk samples without the need for complex or time-consuming pre-treatment steps. The measurement principle was based on the competitive immunological reaction and silver enhancement technique. In this case, a visible array dots as the detectable signals were further amplified and developed by the silver enhancement reagents. The microarray could be assayed by the microarray scanner. The detection limits (S/*N* = 3) were estimated to be 40 ng/mL (α-LA), 50 ng/mL (β-LG), 30 ng/mL (LF) (*n* = 6).

**Conclusions:**

The method could be used to simultaneously analyze the whey protein contents of various raw milk samples and ultra-high temperature treated (UHT) milk samples including skimmed milk and high calcium milk. The analytical results were in good agreement with that of the high performance liquid chromatography. The presented visualized microarray has showed its advantages such as high-throughput, specificity, sensitivity and cost-effective for analysis of various milk samples.

**Electronic supplementary material:**

The online version of this article (doi:10.1186/s12896-017-0387-9) contains supplementary material, which is available to authorized users.

## Background

Milk whey protein represents a rich and mixture proteins with wide ranging nutritional, biological and food functional attributes. The main constituents are α-lactalbumin (α-LA), β-lactoglobulin (β-LG) and lactoferrin (LF), which account for approximately 70–80% of total whey protein. α-LA, β-LG and LF are of high nutritional value which have made ingredients of choice in the formulation of modern foods and beverages. They may also have physiological activity through moderating gut microflora, mineral absorption and immune function [1, 2].

Although several methods have been reported for α-LA, β-LG and LF, either alone or concomitant with other whey proteins, including chromatographic analysis (High performance liquid chromatography (HPLC) [3–11], Ultra high performance liquid chromatography (UHPLC) [12], High performance liquid chromatography -mass spectra (HPLC-MS) [13–21], Ultra high performance liquid chromatography - mass spectra (UHPLC-MS) [22–27], Immunoaffinity chromatography (IAC) [26, 27]), Radial Immunodiffusion (RID) [28], sodium dodecyl sulfate polyacrylamide gel electropheresis (SDS-PAGE) [29, 30], Capillary Electrophoresis(CE) [10, 31–34], Enzyme-llinked Immunosorbent Assay (ELISA) [17, 35–42], Fluorescent Immunosorbent Assay(FIA) [43, 44], Surface Plasmon Resonance (SPR) [45–49] and Sensors [50–52]. In general, chromatographic analysis requires pre-treated samples, high initial sample volumes and long analysis times, which lead to high cost. In addition, analytical chromatographic technologies are unable to identify protein denaturation or modification that may occur during processing and storage. This is an important factor for public health and food commodities marketing. Some of these drawbacks can be overcome using traditional immunological methods, such as ELISA. It also offers the advantages of working directly with complex fluids, such as whole milk and other dairy fluids, but only one whey protein can be detected. However, there remains an urgent need to develop alternative methods for quantification featuring reduced cost, improved sensitivity, selectivity and more rapid response, especially for simultaneous detection of multiple whey proteins.

Development of new tools, minimizing limitations imposed by these methodologies and leveraging the high specificity of traditional immunological methods, is of great interest. In this sense, visualized microarray are envisaged as a valid alternative to classical methods for analysis of protein, because they are amenable to direct readout by eyes and well suited to rapid detection with high sensitivity and selectivity using low-cost instrumentation that is adaptable to portable, field-deployable embodiments, which is ideal for routine determination in the dairy industry [53–56].

In this paper, we described the development of visualized microarray method for simultaneous, high-throughput quantitative immune-detection of three commercially important whey proteins (α-LA, β-LG, and LF) in samples at a time, from various milk sources. To the best of our knowledge, no visualized microarray has been described thus far for the determination of a-LA, β-LG, and LF simultaneously. Visualized microarray method allowed the analysis of milk without the need for sample preparation, including pre-enrichment or purification steps, “extraction” of target analytes from the complex matrix, and measurement of signal in a “clean” environment. The assay was then used to simultaneously analyze the whey protein contents of various raw milk samples and UHT milk samples including skimmed milk and high calcium milk and the analytical results were in good agreement with that of the HPLC.

## Methods

### Materials and instruments

α-LA, β-LG, LF and silver enhancement solution including solution A (AgNO_3_) and solution B (Hydroquinone) were all purchased from Sigma-Aldrich. NaCl, KCl, Na_2_HPO_4_·12H_2_O, KH_2_PO_4_, Tween-20, Ethylenediaminetetraacetic acid (EDTA) was from Nanjing Chemical Reagent Co., Ltd. (Nanjing, China). Pure water of 18.2 MΩcm-1 was generated in-lab from a Milli-Q water system. Bovine serum albumin (BSA) was purchased from Merck. Goat polyclonal to α-lactalbumin (α-LA), goat polyclonal to β-lactoglobulin (β-LG), goat polyclonal to lactoferrin (LF) and AgNPs labeled donkey anti-goat IgG were kindly supplied by Nanjing Xiangzhong Biotechnology Co. Ltd. (Nanjing, China).

All solutions were made by triply deionized water (Milli-Q water purification system, Millipore, Billerica, MA, USA). A 10 mM phosphate buffered saline (PBS) at pH 7.2 was used as the assay buffer which was prepared as following: 137 mmol/L NaCl, 2.7 mmol/L KCl, 10 mmol/L Na_2_HPO_4_·12H_2_O and 2 mmol/L KH_2_PO_4_. A 10 mM PBS containing 0.01% Tween-20 and 1 mM EDTA (PBST- EDTA) at pH 7.2 was used for milk sample preparation and dilution. The wash buffer was a PBS containing 0.05% Tween 20 (PBST). The blocking solution was 1% BSA in 10 mM PBS. All buffers were filtered through 0.22 μm pore size filter before use.

The microarrays were prepared by TMAR microarray spotter (Tsinghua University, Beijing, China). Automated plate washer (BioTek Instruments, Inc. America) was used as washing platform. LXJ-II centrifuge (Shanghai Anting Instrument Co., Shanghai, China) were used for the centrifugation. Clear flat-bottom 96-well plate, thermo-shaker and microarrays scanner (QARRAY 2000) were from Nanjing Xiangzhong Biotechnology Co. Ltd. (Nanjing, China).

### Microarray preparation

The obtained of α-LA, β-LG and LF were spotted on clear flat-bottom 96-well plate. A volume of 10 μL of each coating antigens diluted by spotting buffer were arrayed with a 500 μm spot-to-spot pitch using a microarray spotter, each antigen solutions was in triplicate. After spotted, microarray was incubated for 2 h at 37 °C. In this step the coating antigens were immobilized on the microplate wells by absorption over the surface of the support of polystyrene. After immobilization, microarray surface was treated with 200 μL 1% BSA for 1 h at 37 °C in order to minimize further unspecific bindings. After incubation, the microarray plate was washed with 1 × PBST buffer using an automated plate washer and then sealed in foil packets for storage at 2–8 °C.

### Indirect competitive microarray immunoassay protocol

**Scheme 1:**
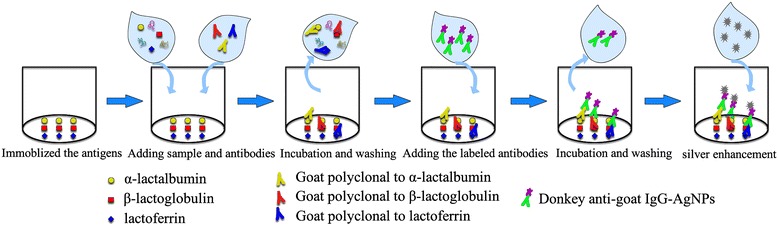
Schematic illustration of detection α-lactalbumin (α-LA), β-lactoglobulin (β-LG) and lactoferrin(LF) with visualized microarray immunoassay platform, composed of silver enhancement amplification system

The indirect competitive microarray immunoassay principle was presented in Scheme 1. In a microarray immunoassay analysis the following experimental procedure was performed. The competition is established by the addition of a mixture of 25 μL the standard (or the sample), a known amount of 25 μL mixed antibodies, The reaction is incubated at 25 °C for 45 min on a thermoshaker (shaking at 600 rpm). After the corresponding washing step, AgNPs labeled donkey anti-goat IgG in a total volume of 50 μL/well. The reaction is incubated at 37 °C for 30 min on a thermoshaker (shaking at 600 rpm). After the corresponding washing step, 50 μL silver enhancement solution including solution A (AgNO_3_) and solution B (Hydroquinone) was then added to each well, and incubated for 12 min at 37 °C in dark. At the end of colorimetric reaction, each well was washed 3 times with 250 μL pure water.

### Microarray imaging and data processing

The microarray was imaged with microarray scanner (QARRAY 2000) and performed using the corresponding software to quantify the signal over the sample spot area and expressed as relative light units (RLUs). The calibration curve was represented by a linear relationship.

### Cross reactivity calculation

Cross reactivity (CR) is generally defined as the necessary amount of mass or concentration of interference able to produce an equal signal as when the analyte is assayed to provoke a signal inhibition of 50%. Therefore, in this work CR rates, in terms of percentage (%), were calculated according to the expression (eq. 1).

1 $$ \mathrm{CR}=\left[\left({\mathrm{IC}}_{50}\left(\mathrm{analyte}\right)/{\mathrm{IC}}_{50}\left(\mathrm{interference}\right)\right)\right]\times 100\% $$

IC_50_ is the necessary concentration of analyte or interference to induce a signal inhibition of 50%.

### Milk samples

Milk consists of metal ions such as calcium, iron, magnesium and zinc. For actual sample analysis, it should be considered that α-LA, β-LG and LF had a high possibility of forming chelation complex with these metal ions. Thus, prior to actual sample analysis, milk was diluted 200-fold with PBST-EDTA at pH 7.2. Milk was purchased from local supermarket.

### HPLC method

#### Solutions

Binding buffer (BB): 1.211 g Tris was dissolved with 800 mL 6 mol/L HCl, adjusted to pH 7.4 and then volumed to final volume to 1000 mL.

Elution buffer (EB): 0.15 mol/L sodium phosphate, pH 12.

Buffer for adjusting pH of EB (AB): 1 mol/L sodium dihydrogen phosphate.

#### Treatment of milk sample for analysis α- Lactalbumin and β- lactoglobulin

5 mL milk sample was mixed with 14 mL water and adjusted to pH 4.6. Next water was added to the mixture making final volume to 20 mL. Then the above mixture was centrifugated under 10,000 rpm and 4 °C for 10 min. Finally, supernatant was filter with 0.22 μm filter and injected to HPLC system.

#### Treatment of milk sample for analysis lactoferrin

Milk samples were centrifugated under 8000 g and 4 °C for 10 min to remove fat. Then 15 mL skim milk was loaded onto lactoferrin immune-affinity column that was pre-equilibrated with 10 mL BB. After washing with 20 mL BB, lactoferrin was eluted with 3.6 mL EB. Then the 3.6 mL elution was mixed with 0.4 mL EB. Finally the mixture was filtered with 0.22 μm filter and injected to HPLC system. The lactoferrin immune-affinity column was washed with 10 mL BB and stored at 4 °C for further use.

### HPLC system

The chromatographic analysis of lactoferrin was carried out on a HPLC system (2695 Separations Module, Waters; Milford, MA, USA) coupled with a photodiode array detector (PDA 2996 detector, Waters; Milford, MA, USA). Separation was performed using a Symmetry C4 Column (300 Å, 5 μm, 4.6 mm × 250 mm, Waters). Acetonitrile (eluent A) and 0.1% trifluoroacetic acid in water (eluent B) were used as mobile phase. The flow rate was set at 1.0 mL/min and the LC elution gradient was as follows: initial 30% A, 5 min 55% A, 10 min 60% A, 12 min 30% A and hold on for a further 4 min for re-equilibration, giving a total run time 16 min. The column temperature was kept at 25 °C and the injection volume was 50 μL for standards and sample solutions. The wavelengths was set at 280 nm for detection. Waters Empower 2.0 chromatography software package was used for HPLC system control, data acquisition and management.

## Results and discussion

### Optimization

To develop a highly sensitive and specific indirect competitive immunoassay, the conditions including the concentrations of coating antigens and antibodies, should be carefully optimized by a checkboard titration of antigen and antibody simultaneously. In addition, it was necessary to evaluate the effect of presence or absence of EDTA and Tween 20 in assay buffer.

### Concentrations of coating antigens and antibodies

**Fig. 1:**
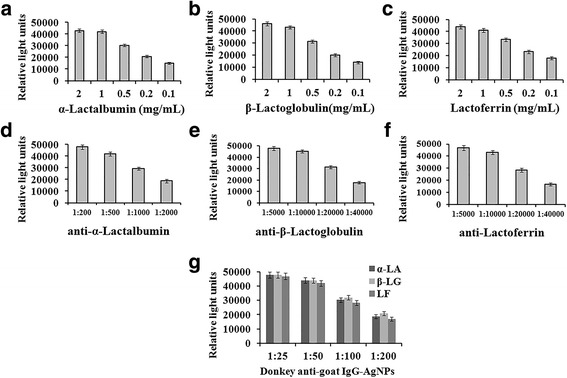
**a**, **b**, **c** coating antigens of α-LA, β-LG and LF were 2 mg/mL, 1 mg/mL, 0.5 mg/mL, 0.2 mg/mL, 0.1 mg/mL, respectively; **d** anti-α-LA were 1:200, 1:500, 1:1000, 1:2000 dilution respectively; **e** anti-β-LG were 1:5000, 1:10,000, 1:20,000, 1:40,000 dilution respectively; **f** anti-LF were 1:5000, 1:10,000, 1:20,000, 1:40,000 dilution respectively; **g** second antibodies of AgNPs labeled donkey anti-goat IgG were 1:25, 1:50, 1:100, 1:200 dilution respectively for α-LA, β-LG and LF

To develop highly sensitive competitive immunoassay, the conditions including the concentrations of coating antigens and dilutions of antibodies should be carefully optimized. In this study, coating antigens of α-LA, β-LG and LF all were 2 mg/mL, 1 mg/mL, 0.5 mg/mL, 0.2 mg/mL, 0.1 mg/mL, respectively; anti-α-LA were 1:200, 1:500, 1:1000, 1:2000 dilution respectively; anti-β-LG were 1:5000, 1:10,000, 1:20,000, 1:40,000 dilution respectively; anti-LF were 1:5000, 1:10,000, 1:20,000, 1:40,000 dilution respectively. In addition, second antibodies of AgNPs labeled donkey anti-goat IgG were 1:25, 1:50, 1:100, 1:200 dilution respectively. The results can be seen in Fig. 1.

Lower the concentration of antigen and antibody can increase detection sensitivity, but the signal value will be lower. So the optimal assay conditions were as follows: Appropriate concentrations of α-LA, β-LG and LF were all 1 mg/mL; The appropriate concentrations of anti-α-LA, anti-β-LG and anti-LF were 1:500, 1:10,000 and 1:10,000 dilution respectively; Appropriate concentrations of second antibodies of AgNPs labeled donkey anti-goat IgG were 1:50 dilution.

### Effect of EDTA and Tween 20

However, milk has metal ions such as calcium, iron, magnesium, potassium, sodium and zinc ion, so it is necessary to consider the high potential for forming chelating complexes between α-LA, β-LG and LF with these metal ions. To prevent these interferences, EDTA was incorporated in the assay buffer. EDTA has a greater affinity for the calciums than α-LA, β-LG and LF, thus it can block the interaction of α-LA, β-LG, LF with calciums.

**Fig. 2:**
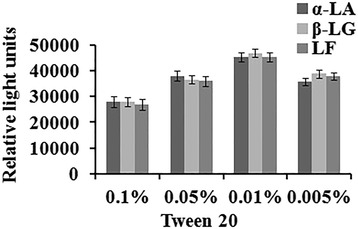
The dilutions of Tween 20 were 0.1%, 0.05%, 0.01%, 0.005% respectively for α-LA, β-LG and LF. Antigen of α-LA, β-LG and LF were all 1 mg/mL; anti-α-LA, anti-β-LG and anti-LF were 1:500, 1:10,000 and 1:10,000 dilution respectively; Second antibodies of AgNPs labeled donkey anti-goat IgG 1:50 dilution

Tween 20 is a non-ionic surfactant, which has emulsification, diffusion, solubilization, stabilizing effect with samples. Moreover, it provides a protective of antigen-antibody in buffers, and reduces the nonspecific binding of antibodies to antigens and interfering proteins. Thus it can reduce the background and improve the sensitivity. However, an excessive concentration could inhibit binding of antibody and antigen. Finally, Tween 20 concentration was selected to be 0.01%. The results can be seen in Fig. 2.

### Method development

**Fig. 3:**
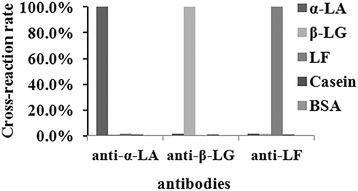
Anti-α-LA, anti-β-LG and anti-LF were cross-reactivity with α-LA, β-LG, LF, Casein and BSA

Assay specificity indicates the ability of antibody to generate a measurable response only for the target molecule. The cross-reactivity of antibodies was evaluated under indirect competitive immunoassay conditions in order to confirm specificity. Here, a study was performed using five main proteins in milk, such as α-LA, β-LG, LF, Casein and BSA. The cross-reactivity studies were carried out by adding various free cross reactants at different concentrations to compete with antigen coated on the surface, to bind with the antibody. The cross-reactivity for each compound was calculated according to the expression (eq. 1) and given in Fig. 3.

The anti-α-LA, anti-β-LG, anti-LF were determined to be highly specific for α-LA, β-LG, LF respectively, although there was a minor dose–response relationship for Casein and BSA (cross-reactivity <1.0%), the binding responses for these proteins were analytically insignificant at concentrations equivalent to those of diluted milk samples.

### Method performance

In order to be able to determine multiplex format concentrations of α-LA, β-LG and LF, the assay was calibrated independently using a cocktail of the α-LA, β-LG, LF antibodies and different concentrations of α-LA, β-LG and LF. As a matter of fact, the competition occurs for all target molecules and the specific signal obtained on each probe decreases with the analyte concentration, as expected in a competitive immunoassay.

**Fig. 4:**
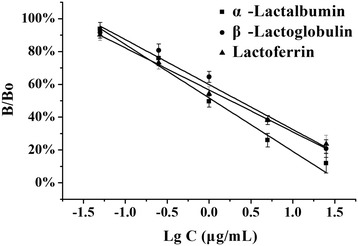
Calibration curves of α-LA, β-LG and LF. α-LA: y = −0.3258× + 0.5171, *r* = 0.9829; β-LG: y = −0.2738× + 0.5986, *r* = 0.9702; LF: y = −0.2558× + 0.5658, *r* = 0.9952

Over the optimized working calibration range (α-LA, β-LG and LF were all 0.05, 0.25, 1, 5, 25 μg/mL), a semi-log curve fit adequately described the dose-response relationship which can be seen in Fig. 4. Their calibration curves were calculated as follows, α-LA: y = −0.3258× + 0.5171, *r* = 0.9829; β-LG: y = −0.2738× + 0.5986, *r* = 0.9702; LF: y = −0.2558× + 0.5658, *r* = 0.9952. In the calculation formula, y: B/B0%, x: lg C. (B/B0 is the ratio of response B to the maximum response when no analyte is present B0.)

The method detection limits (response 3 standard deviations of blank over several independent runs) were estimated to be 0.04 μg/mL (α-LA), 0.05 μg/mL (β-LG), 0.03 μg/mL (LF) (*n* = 6). Method precision was estimated from the aggregate of a single-level control α-LA (1 μg/mL), β-LG (1 μg/mL), LF (1 μg/mL) over multiple independent runs, and the measured RSD were 6.71%, 7.82%, 5.13%, respectively(*n* = 6). Between-run precision may be further assessed with RSD 12.31%, 13.52%, 14.15%, respectively (*n* = 6).

After a simple dilution of commercial milk (200-fold in PBST-EDTA, pH 7.2), use this calibration curve to calculate the concentration of milks. The recovery study was performed samples of milk purchased from local supermarkets. Free α-LA, β-LG and LF (20 μg L^−1^, 100 μg L^−1^, 400 μg L^−1^and 2000 μg L^−1^) were spiked in milk solution. The recovery study was performed in three replicates and the results were quite satisfactory as seen in Table 1.

**Table 1:** The recoveries of different concentrations of α-Lactalbumin, β-Lactoglobulin, Lactoferrin

Proteins	Spiked concentration (μg/mL)	Average (μg/mL)	SD (μg/mL)	RSD	recovery
α-Lactalbumin	20	18.23	2.14	11.74%	91.15%
	100	108.19	12.27	11.34%	108.19%
	400	409.81	40.57	9.90%	102.45%
	2000	2032.93	252.61	12.43%	101.65%
β-Lactoglobulin	20	19.13	2.56	13.38%	95.65%
	100	105.77	11.76	11.12%	105.77%
	400	419.8	48.36	11.52%	104.95%
	2000	2131.46	206.85	9.70%	106.57%
Lactoferrin	20	19.13	2.76	14.43%	95.65%
	100	95.77	12.08	12.61%	95.77%
	400	402.81	40.36	10.02%	100.70%
	2000	1931.46	263.44	13.64%	96.57%

Recovery = (C1-C2)/C3 × 100%

C1: Sample concentration after adding standard.

C2: Sample concentration before adding standard.

C3: concentration of adding standard.

### Comparison with a reference method-HPLC

**Fig. 5:**
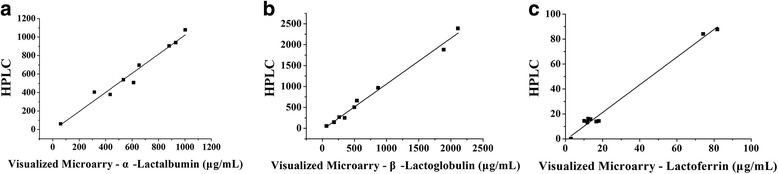
Results obtained by visualized microarray and HPLC are plotted against each other. **﻿a**﻿ α-Lactalbumin, **b** β-Lactoglobulin, **c** Lactoferrin

To verify the reliance and accuracy of visualized microarray system, the results of 9 milk samples were compared with an HPLC method. The results obtained by visualized microarray and HPLC are plotted against each other in Fig. 5. The correlation index r was very good with a linear regression curve of y = 1.031×-9.30, *r* = 0.9604 (α-Lactalbumin); y = 1.094×-35.33, *r* = 0.9872 (β-Lactoglobulin); y = 1.1096×-1.054, *r* = 0.9889 (Lactoferrin); These results confirm those of the validation experiments. The findings indicate that reliable results can be obtained over the whole concentration range.

### Method applications

**Fig. 6:**
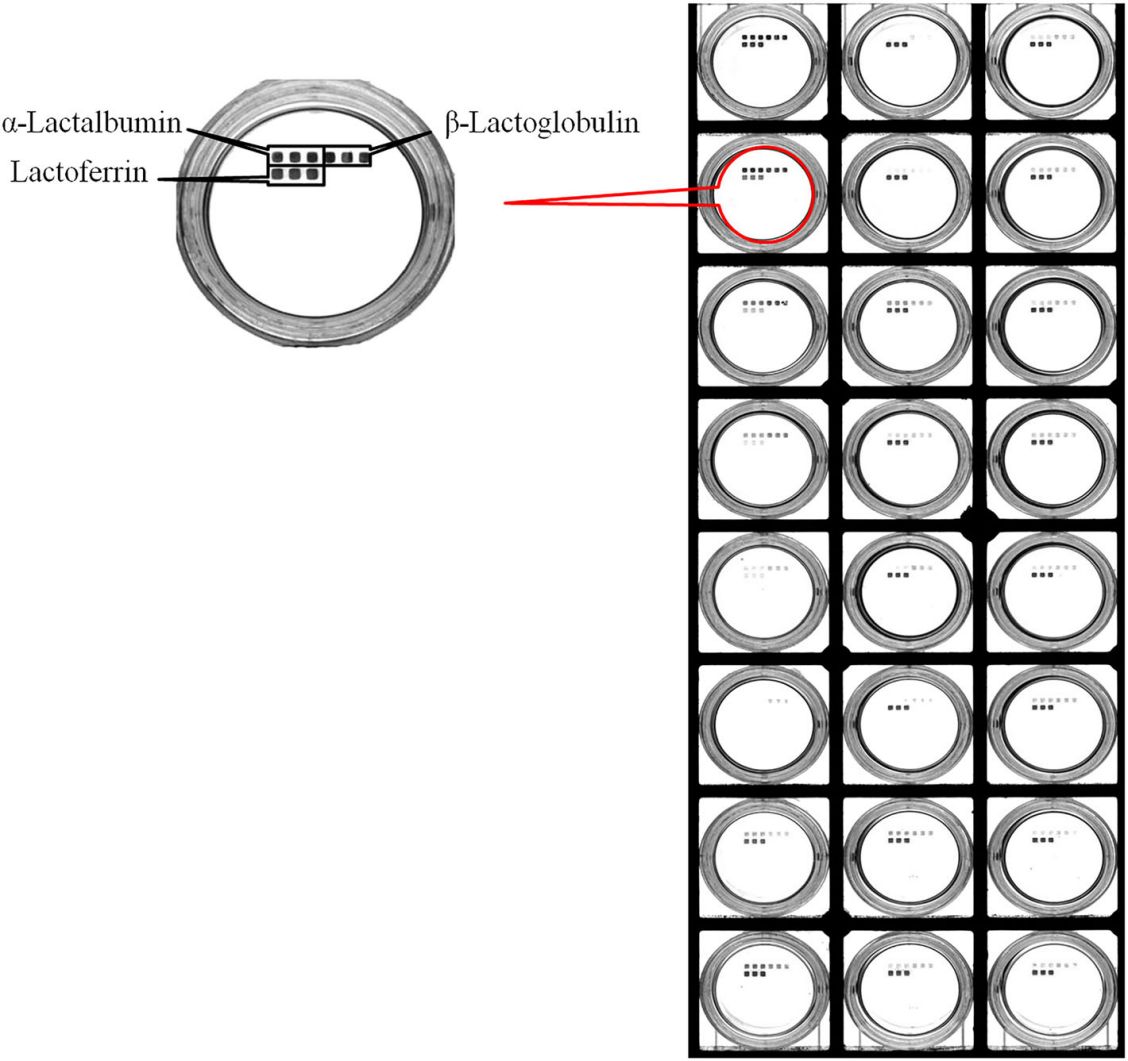
Results of α-LA, β-LG, and LF were detected by Visualized Microarray. From top to bottom, left to right was numbered 1 to 18. 1–7 were raw milk, 8–11 were pasteurized milk, 12–18 were UHT milk including skimmed milk and high calcium milk

The developed procedure was then applied to quantify the concentration of native α-LA, β-LG and LF in three different kinds of milks. The results were shown in Additional file 1﻿, Fig. 6 and Table 2. The precision of the results were well (RSD < 15%). As samples, two bovine milks with different processing treatments have been analyzed. Taking into account the calibration curve, it has been determined that raw milk which numbered 1–7 presented highest concentration of α-LA, β-LG and LF,then pasteurized milk (72–85 °C for 15 s) which numbered 8–11, UHT milk (135–150 °C for 4–15 s) including skimmed milk and high calcium milk which numbered 12–18. As compared to other references that mention the concentration of α-LA, β-LG and LF in milk [12, 34, 50]. Now, it is well known that α-LA, β-LG and LF were highly sensitive to temperature.

**Table 2:** Results of α-LA, β-LG, and LF detected by Visualized Microarray

Proteins	number	α-Lactalbumin			β-Lactoglobulin			Lactoferrin		
		Average (μg/mL)	SD (μg/mL)	RSD (*n* = 3)	Average (μg/mL)	SD (μg/mL)	RSD (*n* = 3)	Average (μg/mL)	SD (μg/mL)	RSD (*n* = 3)
Raw Milk	1	230.53	21.79	9.45%	490.20	60.93	12.43%	124.2	13.23	10.65%
	2	80.86	8.36	10.34%	70.70	8.29	11.73%	11.8	1.44	12.21%
	3	1315.13	130.20	9.90%	983.09	104.01	10.58%	6.1	0.69	11.23%
	4	653.01	79.99	12.25%	964.84	139.42	14.45%	7.9	1.01	12.78%
	5	150.21	16.88	11.24%	211.13	27.68	13.11%	28.5	3.62	12.69%
	6	454.64	38.83	8.54%	440.27	41.17	9.35%	10.3	1.12	10.88%
	7	811.91	107.82	13.28%	348.66	44.84	12.86%	21.0	3.10	14.78%
pasteurized milk	8	534.7	75.45	14.11%	499.84	67.23	13.45%	18.7	2.58	13.80%
	9	199.45	19.89	9.97%	166.57	17.79	10.68%	23.6	2.34	9.93%
	10	461.9	54.23	11.74%	308.7	46.12	14.94%	15.6	2.00	12.81%
	11	409.4	58.83	14.37%	243.6	32.57	13.37%	0.0	-	-
UHT Milk	12	263.6	34.00	12.90%	229.5	27.88	12.15%	0.0	-	-
	13	348.2	46.87	13.46%	205.5	29.65	14.43%	0.0	-	-
	14	264.3	33.51	12.68%	307.6	31.87	10.36%	10.2	1.26	12.39%
	15	503.0	66.30	13.18%	327.4	39.68	12.12%	6.7	0.82	12.17%
	16	251.3	35.73	14.22%	169.6	23.10	13.62%	8.1	1.01	12.51%
	17	579.6	60.57	10.45%	737.0	97.21	13.19%	12.4	1.43	11.56%
	18	312.8	34.10	10.90%	255.5	30.02	11.75%	0.0	-	-

## Conclusions

In this work, visualized microarray for the high-throughput, specific and sensitive determination of a-LA, β-LG and LF in milk samples was developed for the first time, without the need for complex or time-consuming pre-treatment steps, following dilution with an appropriate working buffer. The applicability of the visualized microarray as-developed was underlined by the implementation and analysis of different milk samples, and the results were validated successfully against a HPLC. The visualized microarray performance is in accordance with such an ELISA kit in terms of rapidity, sensitivity, simplicity and inexpensive, However, ELISA detect α-lactoalbumin, β-lactoglobulin and lactoferrin in milk, it needs at least three times of experiments. Therefore, it has potential as an alternative analytical tool to screen for the presence of a-LA, β-LG and LF in the dairy industry and pediatric foods. Moreover, the implementation of disposable conjunction with the simplicity, automation and miniaturization of the instrumentation constitute important advantages leading towards the integration of the method in portable (in-field), reliable and user-friendly analytical systems for milk and infant formula quality control.

## Additional file

Additional file 1: Figure S1. The microarray of α-LA, β-LG, and LF on clear flat-bottom 96-well plate after silver enhancement was imaged with microarray scanner (QARRAY 2000). From top to bottom, left to right was numbered 1 to 18. 1–7 were raw milk, 8–11 were pasteurized milk, 12–18 were UHT milk including skimmed milk and high calcium milk. (JPEG 3520 kb)

## Abbreviations

AB: Buffer for adjusting pH of EB
a-LA: α-Lactalbumin
BB: Binding buffer
BSA: Bovine serum albumin
CE: Capillary Electrophoresis
CR: Cross reactivity
EB: Elution buffer
EDTA: Ethylenediaminetetraacetic acid
ELISA: Enzyme-linked immunosorbent assay
FIA: Fluorescent Immunosorbent Assay
HPLC: High performance liquid chromatography
HPLC-MS: High performance liquid chromatography -mass spectra
IAC: Immunoaffinity chromatography
LF: Lactoferrin
PBS: Phosphate buffered saline
RID: Radial Immunodiffusion
RLUs: Relative light units
SDS-PAGE: sodium dodecyl sulfate polyacrylamide gel electrophoresis
SPR: Surface Plasmon Resonance
UHPLC: Ultra high performance liquid chromatography
UHPLC-MS: ultra high performance liquid chromatography - mass spectra
UHT: Ultra-high temperature treated
β-LG: β-lactoglobulin
