# Supplementary figures and images for: Simultaneous detection of α-Lactoalbumin, β-Lactoglobulin and Lactoferrin in milk by Visualized Microarray
Source: BMC Biotechnol. 2017 Sep 12;17:72. doi: 10.1186/s12896-017-0387-9 (PMC5596914; doi:10.1186/s12896-017-0387-9)

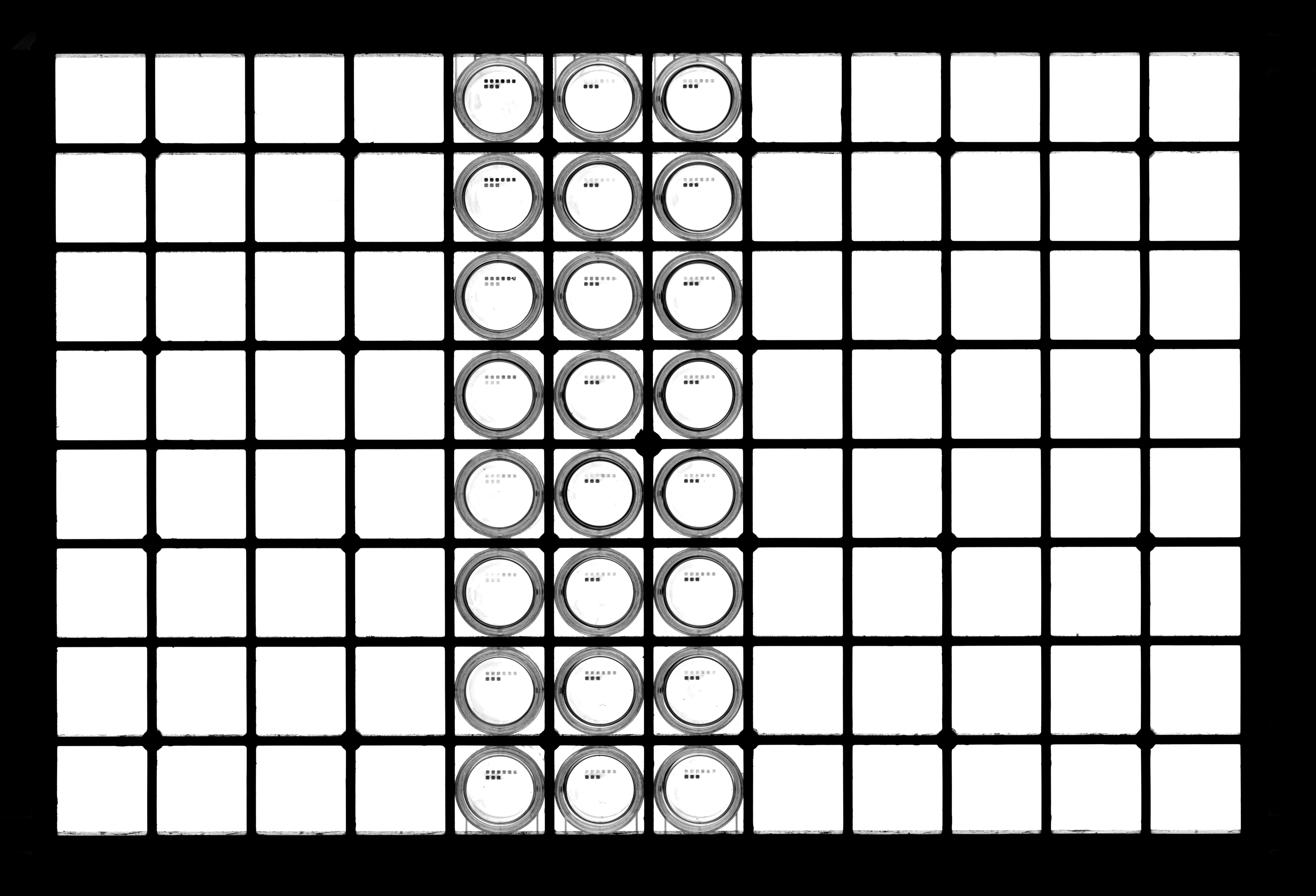

Supplement: Additional file 1: Figure S1. — The microarray of α-LA, β-LG, and LF on clear flat-bottom 96-well plate after silver enhancement was imaged with microarray scanner (QARRAY 2000). From top to bottom, left to right was numbered 1 to 18. 1–7 were raw milk, 8–11 were pasteurized milk, 12–18 were UHT milk including skimmed milk and high calcium milk. (JPEG 3520 kb) [file 12896_2017_387_MOESM1_ESM.jpg]
